# A Report of Cases of Abdominal Surgery*Read before the North Texas Medical Association, December 11, 1902.

**Published:** 1903-01

**Authors:** Henry K. Leake

**Affiliations:** Dallas, Texas


For Texas Medical Journal.

### A Report of Cases of Abdominal Surgery.*

BY HENRY K. LEAKE, M. D., DALLAS, TEXAS.

I begin this paper with an apology for its incompleteness. The substance of a remark made by Ruskin is that in these days what we most need are facts, and facts that should be told in a downright, plain way. By following this advice in an attempt to narrate some clinical facts, that at least may re-establish your thoughts along certain lines not yet exhausted, I hope to atone somewhat for the absence of scientific details that might render this paper more acceptable.

We are all getting to be surgeons; probably never before in the history of the profession did surgery offer more fascination for the medical man than it does at the present time, although among the ancient Egyptians, Greeks and Romans the great surgeons of the schools were then, as now, the magnets that attracted the most attention from the laity and the students. During the middle ages—except at Salerno, in Spain—surgery, like medicine, had few devotees, owing to proscriptive laws and the chaotic social conditions; but from the fifteenth century until the dawning of the antiseptic era of surgery the enthusiasm for surgery over that of medicine revived, and, with a slight increase perhaps, maintained a degree alike with that that possessed the minds of men before the medieval period. Since the advent of antiseptic surgery, surgical ardor has reached fever heat, and not to be a surgeon is imagined to endure a sort of opprobrium that is humilating, if not degrading, to the average medical man. There are worlds to conquer in the medical field, but the invading and triumphal heroes are few. The great army medical seems not encouraged by the prospect of a victorious occupation. As in the ancient schools of Rome and Alexandria, the medical colleges of our era make a better show of diagnosis and pathology, but these the average student esteems not as an absolute necessity for a broad medical education, but as a passport that he must win to secure an opportunity for obtaining the instruction to be had in the surgical clinics to which

---

*Read before the North Texas Medical Association, December 11, 1902.

he is most attracted for that knowledge in surgical mechanics that he aspires to follow in his after-professional life. The country districts, as well as the cities, are filling up with surgeons; every village and cross-road settlement has more than one man who will operate in any department of the surgical art, and it is surprising what good work they will do. In a village about one hundred miles from Dallas a country surgeon recently performed four abdominal hysterectomies successfully, besides numerous other radical operations. In a town of not more than one thousand population, about sixty miles from Dallas, a friend of mine recently opened the abdomen of a man for a gunshot injury; there were seven perforations of the large and small intestines. The surgeon closed several of the apertures by suture and then resected four inches of the illeum, making an end-to-end anastomosis. His patient promptly recovered. Men all through the country are operating for uterine fibroids, intestinal obstruction, tubal pregnancy, nephrectomy, aneurisms, resection of the large and small joints, and so forth; while the operation for appendicitis is looked upon as a mere bagatelle that any tyro can handle successfully. The radical operation for hernia, too, is not viewed with dismay by the country surgeon. I did an operation for radical cure of hernia in a patient who came from a neighboring town. A surgeon at his home, learning that he was about to leave for Dallas, remarked to him: "Why leave for Dallas to have such an operation?—this is a small matter and we can just as well do it here." Now, the surgeon who made this remark was conscientious and I have confidence in his surgical ability and experience. In the small city of Rochester, Minnesota, the Mayo brothers are doing a marvelous work in surgery. Particularly in gall-stone surgery they are attracting widespread notice and admiration; in fact some surgeons in the large cities are going to Rochester to witness the unrivaled accomplishments, in this operation, of these comparatively unknown surgeons. It is said that in this department their results are the best in the United States; and just as Lawson Tait, in the beginning of his career, was sneered at as being a provincial surgeon, but rose to pre-eminence, it is predicted that the Mayo brothers will be, if they are not already, the highest authority, in our country, in the best methods of operating for the different forms of gall-stone obstruction. Such examples might be multiplied; therefore, the pursuit of surgery, and its successful pursuit, is not peculiar to the cities—it has become almost universal, while the medical diagnostician, pathologist and therapeutist are sadly lacking in evidence.

Now, I shall not discuss the question whether or not this condition of affairs professional is of great advantage either to the laity or to the profession. We must admit, however, that in many circumstances this condition will be justified, and this is taking a broad, philosophical and unselfish view of the matter. On the other hand, what is the future of the so-called specialist in surgery? He can hardly hope to maintain his position of eminence by piling up statistics of certain successful operations that have become the common property of the profession, at least statistics of operations for conditions that heretofore assumed to be radical have been eliminated of their complex elements by the tireless, better educated and aggressive surgeons throughout the country. Such complex elements are now familiar conditions; they are recognized and attacked more frequently, consequently they neither startle nor deter the surgeon. They are seen by the medical man who often visits the clinics of the large cities; in short, the so-called country surgeon closely approximates, if he does not rival, the surgeon of the cities, who presumably has larger opportunities of observation and greater facilities for operating. Notwithstanding all this, exceptional conditions in surgery, both as regard diagnosis and operation, increasingly arise; these exceptions occur so frequently that the surgeon of the city is persuaded to tabulate them and then to formulate conclusions that are of inestimable value. They virtually become common conditions in a large experience and a faithful study of them will enable the surgeon of the cities to keep in the forefront of surgical knowledge and teaching. To the surgeon fortunately situated in these respects, this is a great privilege that not only should be exercised modestly, but utilized for the benefit of others of a more limited experience.

The present paper is intended to deal with certain of these exceptions,not exceptions so much as to surgical diagnosis,but those exemplifying methods of operating in special conditions. I am reminded that the title of this paper is somewhat misleading. In Plutarch's Lives are recorded the comparison and contrasts in the lives of certain great men of antiquity. The former appertained to the social, intellectual and political qualities of these men, their physical infirmities were only incidentally mentioned, but the medical clinician might write a volume full of practical wisdom arranged somewhat upon the same plan. This idea might be expanded to include large classes of individuals and develop truths of far-reaching importance to the medical profession. But I shall have to take humbler ground and narrate a few surgical examples presenting marked contrasts, but these perhaps limited in their study and application. On this account they are none the less apparent and instructive; consequently my paper might have been entitled: "Contrasts in Surgical Practice," or words to that effect.

My first surgical contrasts will re-open a well-worn theme in this society—the surgical treatment of appendicitis. They consist of four selected cases, two of them relating to the management of the stump of the appendix in more or less "clean cases;" the last two will torment you with that Nemesis: What to do in pus cases of appendicitis where there is matting of bowel and intestine and the appendix cannot be readily located?

In July last, I was applied to by the son, eighteen years old, of my distinguished friend, Dr. Hadra of this city, for the removal of his appendix that had given him more or less trouble for several months. He had had an attack of acute appendicitis several months previously. There was tenderness with slight rigidity on deep pressure over McBurney's point. He complained of lameness on that side. Four days thereafter, at St. Paul's Sanitarium, the abdomen was opened by a three-inch incision; there were no adhesions; the appendix was long and lay from right to left, closely hugging the head of the cœcum, by a very short meso-appendix that apparently produced an angulated condition of the appendix at its base, thus preventing free drainage. The meso-appendix was tied off in sections and the appendix raised vertically from its bed. It was constricted by a fine silk ligature about one-sixth of an inch from the cœcum and cut away. The stump was touched with pure carbolic acid; a purse-string suture of silk, penetrating the sero-muscular coat of the cœcum, was made to encircle the stump about one-half inch distant; this was drawn taut and tied, inverting the stump within the cœcal walls. Aside from slight pains the morning succeeding the operation, his recovery was uneventful; he left the hospital for home on the eleventh day. He has called upon me once since to say that he feels perfectly well.

Dr. Hadra objected to the purse-string suture as applied to his son's case. He claimed that the tension would be great enough, probably, to cause strangulation of the circulation of the bowel at that point and thus favor sloughing. This contention is logical, but in my experience it has proved theoretical, for a similar procedure in other cases has not proved disastrous—the suture never has been heard from.

The purse-string suture for closing a round hole in the bowel of not too large dimensions is an ideal method of accurately co-apting the serous coats that will seal the opening; such a suture has found a useful application in bullet wounds of the intestine. Applied at much distance from the base of an appendix, thus constricting a large extent of surface, it may be objectionable. Retaining my confidence in the purse-string suture, this objection has been overcome (Price), as was done in the following case:

On November 14, 1902, at St. Paul's Sanitarium, I operated for my friend, Dr. Allen, in the case of Miss D———. She is eighteen years of age, tall, of spare habit of body. Seven months before she is said to have had an attack of typhoid fever, that lasted six weeks. Menstruation always scant and painful. Since the attack of typhoid fever, that now is suspected of having been appendicitis, she has had several attacks of the latter disease. On examination much tenderness was elicited on firm pressure over and below McBurney's point; *per vaginam* tenderness was found in the region of the right ovary. Diagnosis: ovario-appendicular inflammation. A four-inch incision was made in the right semilunaris. The illeo-cœcal junction of bowel, not adherent, was withdrawn, and the appendix, four inches in length, rigid and enlarged, presented, exactly as in the case above described. The long and thick meso-appendix was tied off and dissected down to the surface of the cœcum. The assistant held the appendix taut, perpendicular to the cœcum. Two long straight needles threaded with silk were passed on each side through the base of the appendix and through only the serous and muscular coats of the organ. The appendix was cut away an eighth of an inch above the needles, after which the needles were pulled through, leaving two long ends of thread on both sides of the stump. These threads were made taut by myself and the assistant and tied down on the face of the stump, the assistant, meanwhile, turning in the serous coat and depressing the mucous coat by a small pair of forceps. It was observed now that the serous coat had involuted "like the petals of a rose," the opening had been firmly closed, the stump of the appendix presenting very much the appearance of the small end of a cigar. The mucus lining had receded within the lumen of the cœcum and the entire stump of the appendix presented in a slight concavity. The cut meso-appendix was drawn up over this concavity and sowed like a graft upon the surface of the bowel, thus affording perfect security against possible leakage and adhesion of neighboring structures to the amputated surface of the meso-appendix. The appendix was enlarged by chronic interstitial inflammation, had a narrow lumen and a constriction near its tip. With its tube the ovary was removed; it had not been adherent to the appendix, but was much enlarged by chronic inflammation, its tunic being much thickened and a small hematoma existing near its surface. The patient recovered and left the hospital at the end of four weeks.

The advantages of this method of disposing of the stump of the appendix in suitable cases are obvious, and, in my judgment, cannot be improved upon. It is a marked contrast to all other methods having the same object in view.

In more or less protracted cases of appendicitis, with the formation of an abscess and a mass of adherent bowel and omentum, the appendix not appearing on emptying the abscess, will you remove all the pathology, or as Treves did, in the King's case, simply open the abscess cavity and drain with tube or gauze?

In Deaver's first edition of his monograph on appendicitis, he urges the skilled operator to disinfect the abscess cavity and then invariably to secure and to remove the diseased appendix, but I did not find him taking his own advice always in these cases. At no place has he insisted upon freeing all adhesions of the bowel and omentum and repairing by Lembert suture bowel surfaces injured by this procedure, thus eliminating the entire pathology of the case. But this is the advice of some English surgeons and Joseph Price, who claim that: "The operation is done as much for removing the adhesions as for extirpating the diseased appendix." Such an operation restores the parts to an approximately normal condition, the diseased appendix and the adhesions are removed, thus obviating a secondary operation for the removal of the former and possible bowel obstruction caused by the latter. In several bad cases I have done this operation with the best results, in others much worse I have not ventured to perform it. Observe the two following contrasted examples:

Last July, a young man, S———, twenty-six years of age, of medium build and height, and with a care-worn expression, called at my office and asked for an examination. His sickness had begun six days previously, without vomiting, but with pains radiating over the entire abdomen and finally locatizing in the right iliac region. His temperature was 101°, pulse 100. The abdomen was slightly tympanitic, there were tenderness and rigidity over the appendicular region. His bowels had been moved previously. He was informed that he had appendicitis and should be operated immediately. He demurred to this, averring that he was far from his home in Mississippi and that already he had had enough trouble with an operation for appendicitis—his father having been operated recently in Memphis for the same disease. Two days afterwards he entered St. Paul's Sanitarium and submitted to the operation. Under the anesthetic no well-defined mass could be detected in the appendicular region, over which, however, there was diminished resonance. The abdomen was opened by the curved incision just wtihin the ilium. There were no adhesions to the abdominal parietes, but a mass consisting of adherent bowel and omentum closely glued to the right ilium presented. The appendix was not recognized. The mass was carefully peeled from its connections with the ilium and the layers of the meso-colon broken through by the finger, whereupon several drams of foul pus welled up. The abscess cavity was disinfected with Deaver's solution, all adhesions separated and the ilio-cœcal junction brought without the abdominal cavity. A long, enlarged and perforated appendix, in a semi-necrotic condition, curving downwards over the head of the cœcum, crossing the left leaflet of the meso-cœcum and extending out for several inches upon the ilium, to all of which structures it was densely adherent, came into view. The leaflets of the the meso-cœcum, between which the pus surrounding a concretion, had formed, were in a condition of imminent gangrene and presented as ragged, irregular flaps that had been torn up by the force necessary to eventrate the bowel; altogether the parts looked semi-gangrenous. The appendix was released from its connections to the ilium, meso-cœcum, and cœcum, leaving surfaces torn through the sero-muscular coat of the bowel and oozing freely. It was found possible to invaginate the base of the appendix, the torn flaps of meso-cœcum were trimmed away and all abraded surfaces were tediously repaired with several layers of Lembert sutures of silk and catgut. The parts were irrigated with quantities of hot saline solution and returned within a coffer-dam of gauze, an extra strip being placed near the lines of suturing. The wound was left open and lightly packed with gauze. Recovery was uninterrupted, there being only slight suppuration in the margins of the incision through the abdominal wall. In this case, it will be remarked that the abscess cavity was not so large as is found often in cases similar in other respects, but it is a case, nevertheless, that probably would have been simply drained by the average operator. Narrating the case to Joseph Price, he warmly commended the performance as being truly rational.

Considering the cases with large abscess cavity that, as a rule, I have not ventured to treat by this method, I relate the following history in contrast with the preceding:

S. T., Italian, age thirty-three, short and robust looking. July 10, 1902, I found him in bed with his clothes on; he had been walking around for the nine days that he had been complaining. At no time had there been vomiting, only pain diffused throughout the entire abdomen, that was tender chiefly in the right iliac region where there were also tympany, induration and tumefaction. Pulse 110, temperature 102°. Before going to St. Paul's Sanitarium he went to a barber's shop and was shaved; operation in the afternoon. Incision was made near the ilium; no adhesions to abdominal wall, but there was a huge mass of matted cœcum, omentum and small intestine fixed in the iliac fossa; this mass was of a dark purplish hue. The parts were loosened *en masse* from the iliac fossa and lifted towards the median line, thereupon several ounces of grumous pus, having a strong fecal odor, issued from beneath the cœcum, where was discovered a large abscess cavity having ragged, gangrenous walls, through which there was a hole leading into the bowel. The area of gangrene was large and shaded off into the lateral walls and into the axis of the gut. The appendix could not be located by any justifiable search. It was assumed to have disintegrated into the foul pus present. The abscess cavity was poured full of peroxide of hydrogen and mopped out with Deaver's solution, but no radical measures were taken, either to demonstrate an appendix, or to separate the mass into its primal elements, or to resect adherent or thickened omentum. The abscess cavity under the cœcum was packed loosely with gauze, and the entire diseased mass ensconced within a gauze coffer-dam, shutting off the abdominal cavity. The wound discharged great quantities of feces and pus for eight weeks. During the fifth week a profuse hemorrhage abruptly began in the wound, necessitating quick application of forceps, intravenous transfusion and other measures to save his life. Hemorrhage remained checked for two weeks, when it occurred as before. The deep epigastric artery, that had become necrotic at one point, was tied. Afterwards, the case proceeded satisfactorily. All discharge gradually ceased, the wound closed and the patient made an excellent recovery, without hernial protrusion. In such a case as this, it would seem sheer madness to have proceeded otherwise—but Price and Deaver are past-masters in this department of abdominal surgery.

Disease of the gall tracts afford many antithises in diagnosis and in medical and surgical treatment. I shall recite the brief history of three recent and instructive cases:

W. B., age twenty-nine, tall brunette, sparely built, nervous temperament, moderate drinker. At midnight in his room at a hotel, he was seized with agonizing pain in the region of the stomach; previously his health had been perfect. By attending physicians his suffering was relieved by hypodermics of morphine. Arriving at Dallas he entered St. Paul's Sanitarium. Examination showed a more or less globular tumefaction surmounting the extremity of the ninth rib. This swelling was tender and surrounding it the abdominal muscles were rigid; the stomach was quiet, he was not jaundiced; bowels constipated. The abdominal wall was incised from the ninth rib vertically downwards to a point on a level with the umbilicus. A distended gall bladder suddenly "looked at us;" it was opened and cleared of seventeen gall stones, ranging in size from the end of the thumb to a small pea. They were bile-stained, but there was no bile in the gall bladder. Search downwards for more gall stones was unwisely hurried. The gall bladder was stitched in and drained. Healing of the wound occurred without suppuration. During his three weeks' stay in the hospital no bile was discharged through the rubber tube. He resumed his occupation—traveling salesman—with a sinus discharging a glairy mucus. Several weeks afterwards, he re-entered the hospital; the sinus had not closed and the discharge was irritating the abdominal surface over a wide area. Stricture of the cystic duct was suspected, as in a case reported by Senn, and extirpation of the gall bladder contemplated, but finally the sinus was widely dilated and probed. A stone was felt deep down in the cystic duct; this was extracted with much difficulty and traumatism; a second stone also was removed from the same locality. These stones were of the size of the end of the third finger, and faceted. Two days thereafter, the patient left the hospital, the sinus discharging bile freely; it has now completely closed.

Mr. P., age sixty-two, tall, wiry, in good flesh. For the past eight years he has had attacks of gall-stone colic that interfered seriously with his business as a cattle man in West Texas. No indigestion, bowels regular; no jaundice, abdominal walls lax, no swelling nor tenderness at any point. He was operated at St. Paul's Sanitarium.

The gall bladder was contracted into a body with dense walls, enclosing a cavity holding less than two drams; it was empty, but bile-stained; it was united by strong adhesions to the liver, colon and duodenum. The adhesions to bowel were separated, leaving long rents through the sero-muscular coats. With the finger in the foramen Winslow, the hepaticoduodenal ligament was pulled forward and a large stone found in the cystic duct just above its junction with the hepatic duct. The stone was impacted, it could not be moved; therefore the duct over it was incised and the stone pried out, when bile welled up through the opening. The entire gall bladder was dissected from the liver to a point below the incision in the cystic duct; the latter was tied off with silk. The cut surface on the liver bled freely and was closed with deep sutures of catgut supported by gauze packing. Torn bowel surfaces were united with several lines of Lembert sutures of silk. Through inadvertence, the ligature on the cystic duct was pulled off, when bile freely escaped. The attempt to retie the duct as before was futile, it had retracted; attempts to transfix it with a deep suture line were not satisfactory. A coffer-dam filled with gauze ropes was passed to the bottom of the wound. Reaction was prompt, the temperature and pulse remaining normal during the stay of the patient in the hospital for several weeks. He was discharged with a small sinus emitting moderate quantities of bile.

According to Hadra, Vanderveer, Mayo Robson and others wounds of the gall ducts may be expected to heal kindly if proper drainage be employed; they need not be sutured. Hadra asserts that an opening of the cystic duct, as in my case, will heal as readily as a wound made in the side of the duct to extract a stone, and that, therefore, it was not absolutely necessary to tie the cystic duct. (I may mention that, in 1896, I was compelled to open the duodenum—McBurney—in the case of a patient with a stone in the lower end of the common duct. A large stone was extracted and the duodenal wound sewed up with Lembert sutures. Drainage by the lumber-stab method, of Morrison and Vanderveer, was employed.)

Mrs. J. H., age forty-four, mother of several children, tall, weighs one hundred and sixty pounds. Large amounts of adipose tissue throughout the body, particularly in the abdominal walls. All tissues appear flabby, muscles small. In May, 1902, her sickness began with jaundice and nausea, no colicky pains; she was moderately jaundiced, urine full of bile and urates. She was treated several weeks for catarrh of duodenum and choledecus without effect upon the jaundice and indigestion. She was sent to Mineral Wells, where she drank the waters for several weeks with some benefit as respects the stomach symptoms, but the jaundice persisted and increased through the summer and fall. November 17, 1902, consultation was held with Drs. Pace and Graham. She had lost thirty or forty pounds and was growing weaker. Since the birth of her last child she had experienced intermittent pains, but not severe, in the left lumbo-iliac zone, but on examination no pathology could be discovered here. The stools were ordinarily of a light color. A stone loose in a dilated choledecus, or a malignant growth, was suspected and operation recommended as affording the only hope of reilef. This was done at St. Paul's Sanitarium November 18, 1902, when the gall tracts were examined through a long and deep incision. Owing to the immense amount of fat in the omentum and intestines, together with an apparently small liver deeply situated, the exploration was difficult and prolonged for two hours in an attempt to find the obstructing cause. There were no stones in the ducts, the gall bladder was distended with bile to the amount of two plus ounces; it was opened, and probes passed down to the common duct, which seemed obliterated. No tumor in the head of the pancreas or hilum of the liver or vicinity was detected, consequently there seemed to be no pressure cause. The liver was free from growths, but it was suggested that some division of the hepatic duct in its substance was obstructed by a stone that shut off a large area of secreting lobules, or that there was malignant invasion of the lumen of the duodenum or choledecus (Rolleston) that eluded discovery. Following a general principle in all gall duct obstruction, the gall bladder was stitched to the abdominal parieties and drained. For the first twenty-four hours after the operation, the condition was fairly satisfactory, but now the heart showed signs of failure that progressively increased, until fatal collapse set in on the morning of the third day, notwithstanding intra-venous transfusion, frequent hypodermic strychnia, rectal stimulation and other means usually employed to bring about reaction. There was no evidence whatever of peritonitis. The cause of the chronic jaundice in this case is thus left unknown. The jaundice was pronounced, but the patient was not bronzed as has been seen in other cases. There were no hemorrhages, either from the mucous surfaces, or into the skin; but the vessels in the abdominal incision bled freely, the blood being of a very dark color. Evidently there had been wide-spread alteration in the composition of the blood, the heart muscle, and other organs. Jaundiced patients are notoriously bad subjects for operation, and especially so if such persons, as is often the case, are obese. Murchison gives a limit of three months of persistent jaundice beyond which operations may be expected to prove fatal, but Kehr, Mayo Robson, Dunning and others, have reported successful cases operated upon many months after jaundice had set in from gall-stone obstruction; and I have operated successfully in a case of intense jaundice, with purpuric spots, from this cause that had lasted for nine months, the patient dying from cancer of the liver about six months after the operation.

Concerning the suspicion, mentioned above, that a stone might be present in the common duct, I may remark that the latter condition is not negatived by the fact that there was no colic nor pain precedent to, or coexisting with the jaundice; for, as stated by

Mayo (Stone), a gall-stone nucleus may pass the cystic or hepatic duct without exciting pain. Having lodged in the choledecus, it may acquire great dimensions without any subjective pain indicating its presence.

In long operations which do you prefer, ether or chloroform?

In performing abdominal hysterectomy which is the better method—that with the serre-nœud and transfixion pin, or that after the method of Baer, or one of its modifications?

My last surgical antithsis must leave these problems unsolved:

C. W., Jewess, age thirty-five years, single, weight one hundred and thirty pounds, low stature, ruddy complexion and lively temperament; menses regular, neither dysmenorrhea nor menorrhagia. She had enjoyed good health until last spring, when I attended her for sub-acute rheumatism, lasting several weeks and confining her indoors. During this attack she passed urates in great abundance, but there was neither albumen nor casts in the urine. In midsummer she informed me that she had a lump in the lower abdomen, that she had observed for a year past, and probably for a longer period. It was steadily increasing in size. There were no pressure symptoms. She was greatly perturbed over the situation and demanded removal of the growth. She would accept no delay in the matter. Examination discovered a symmetrical, globular mass, the size of the fifth month of gestation, protruding a thick abdominal wall above the pubis; it was hard and fairly movable. *Per vaginam* no additional information of importance, except that Hegar's sign was absent, was elicited. Stomach quiet, no mammary alterations. Disseminated fibrosis of uterus was diagnosticated, and operation suggested to be done in the fall months. This was declined on the ground that her nervous system would be shattered from the long waiting; other reasons also were urged. Two weeks' delay, in which the system of the patient might be closely investigated, and prepared for the ordeal, was secured. She was ordered warm baths, Buffalo lithia water, laxatives, exercise and nourishing diet without stimulants. The urine was repeatedly examined, and for days before the operation it was judged to be normal in all respects. Operation at St. Paul's Sanitarium on the morning of August 16, 1902; Baer's method selected, the patient being under ether for two hours. She left the operating room in good condition and so remained throughout the day. In the evening the nurse reported that no urine had been passed—there was none in the bladder. Knowing that great care had been taken to isolate the uterine arteries before tying, thus avoiding the ureters, ether nephritis was diagnosticated and every possible means, except intravenous transfusion, were employed to start the kidneys. On the following morning five ounces of smoky urine were collected; it contained eighty per cent of albumen. Treatment was faithfully continued, but no more urine was secreted, death occurring on the evening of the third day. Several hours before death there was emesis and the abdomen swelled rendering it impossible to move the bowels; previously she had passed flatus.

In my next case, I determined to employ chloroform as the anesthetic:

Mrs. P., age thirty-one, farmer's wife, tall, good physique, never pregnant, menses every three weeks and profuse. She had observed a growth in the abdomen for sixteen months past; it was enlarging. Examination showed the physical conditions to be identical with those presented in the case just described. Seven years before, I had curetted this woman for excessive menstruation; the operation failed of its purpose. She entered my private hospital October 6, 1902, and was operated four days thereafter. She inhaled chloroform for an hour and fifty minutes; the uterus and adnexæ being removed by the intra-abdominal method. There was no shock and but little vomiting. For the first twenty-four hours after the operation she passed nine ounces of urine, the second twenty-four hours, sixteen ounces and the third twenty-four hours fifty-two ounces. It contained no albumen and only a small amount of urates. Convalescence was rapid, the patient in fine condition, leaving the hospital at the end of the fourth week, the long incision having united by first intention.

On a recent visit to Northern hospitals, I found that ether was the anesthetic universally given in operations; at all events in adult cases. It is believed to be the safer anesthetic, especially when given in conjunction with oxygen. I saw chloroform administered once only—to a child—and the operator gave it himself with apparent uneasiness. A leading Southern surgeon visiting Philadelphia, told me that he had lost confidence in ether as an anesthetic for the South, his cases did far better with chloroform, other things being equal. Just why there should be this difference in results he could not say, but he had returned to chloroform even in his longest operations; and my inclination is the same. Girdner (Ferrier) asserts that if chloroform kills it is in the beginning of its administration; it seems to make little difference as to the quantity given or the time the patient is under its influence. Undoubtedly, the danger from chloroform arises during its early administration; it is seldom post-operative, as is the case with ether, which more certainly impairs the integrity of the kidneys, although we have a different statement from some writers. For myself, I shall accept the disadvantages of chloroform rather than those of ether, as there appears to be no absolutely certain method by which we may determine the exact condition of the kidneys previously to operation.

I am not sure that abdominal hysterectomy should be performed by the intra-abdominal treatment of the stump, that necessarily entails long and tedious work. Lawson Tait affirmed that a man should complete an abdominal hysterectomy with the serre-nœud in fifteen minutes—I have completed the operation in forty minutes. So far as I know, Joseph Price is the only surgeon in the United States who retains the serre-nœud in his operations. He was taunted with the statement that the intra-abdominal method was more surgical. His reply was: "But what about the saving of human life?" He has finished a series of one hundred and fourteen hysterectomies by the nœud without a death—there was one hernia only. On the other hand, Kelly has done one hundred hysterectomies by the intra-abdominal method, with two deaths; he says nothing about hernias. The operation with the serre-nœud is short, the uterine arteries and ureters are not disturbed, large cellular spaces in the pelvis are not opened up, and Joseph Price says that resulting hernia is a myth.

My foregoing remarks have set forth imperfectly some of the exceptions in the surgical experience of abdominal cases that inevitably will confront the average surgeon. No longer will it avail the so-called surgical specialist to claim that he has performed one hundred appendicitis operations, or fifty abdominal hysterectomies without a death. His methods and results must be reported accurately and truthfully. Moreover, he must not blink the serious cases, or those offering, it may be, a forlorn hope from surgery, all else proving useless. "Playing on the safe side" may enlist the plaudits and support of the laity, but it will not be accepted as a plea for recognition by the average surgeons, who are multiplying with great rapidity, and who will force the surgical specialist of such pretension into a position of mediocrity, above which he may not be able to rise. "To this complexion thou must come at last."

I am indebted to those progressive young surgeons: Drs. Freedman and Fitzpatrick; and to Drs. Whitis and Graham for valuable assistance rendered me in the above reported cases.

